# Comparison of Time Taken to Assess Cognitive Function Using a Fully Immersive and Automated Virtual Reality System vs. the Montreal Cognitive Assessment

**DOI:** 10.3389/fnagi.2021.756891

**Published:** 2021-11-23

**Authors:** Wei Teen Wong, Ngiap Chuan Tan, Jie En Lim, John Carson Allen, Wan Sian Lee, Joanne Hui Min Quah, Muthulakshmi Paulpandi, Tuan Ann Teh, Soon Huat Lim, Rahul Malhotra

**Affiliations:** ^1^SingHealth Polyclinics-Outram, SingHealth Polyclinics, Singapore, Singapore; ^2^Duke-NUS Medical School, Singapore, Singapore; ^3^SingHealth Duke-NUS Family Medicine Academic Clinical Programme, Duke-NUS Medical School, Singapore, Singapore; ^4^Head Office, SingHealth Polyclinics, Singapore, Singapore; ^5^Centre for Quantitative Medicine, Duke-NUS Medical School, Singapore, Singapore; ^6^Technology Development Centre, Institute of Technical Education College West, Singapore, Singapore; ^7^Centre for Ageing Research and Education, Duke-NUS Medical School, Singapore, Singapore; ^8^Health Services and Systems Research, Duke-NUS Medical School, Singapore, Singapore

**Keywords:** cognition, domain, assessment, MoCA = Montreal Cognitive Assessment, virtual reality, dementia

## Abstract

**Introduction:** Dementia is increasingly prevalent globally. Existing questionnaire-based cognitive assessment tools may not comprehensively assess cognitive function and real-time task-performance across all cognitive domains. CAVIRE (Cognitive Assessment by VIrtual REality), a fully immersive virtual reality system incorporating automated audio-visual instructions and a scoring matrix was developed to assess the six cognitive domains, with potential to maintain consistency in execution of the testing environment and possibly time-saving in busy primary care practice.

**Aims:** This is a feasibility study to compare the completion times of the questionnaire-based Montreal Cognitive Assessment (MoCA) and the CAVIRE in cognitively-healthy Asian adults aged between 35 and 74 years, overall, and in and across each 10-year age group (35–44; 45–54; 55–64; 65–74).

**Methods:** A total of 100 participants with a MoCA score of 26 or more were recruited equally into the four 10-year age groups at a primary care clinic in Singapore. Completion time for the MoCA assessment for each participant was recorded. They were assessed using the CAVIRE, comprising 13 segments featuring common everyday activities assessing all six cognitive domains, and the completion time was also recorded through the embedded automated scoring and timing framework.

**Results:** Completion time for CAVIRE as compared to MoCA was significantly (*p* < 0.01) shorter, overall (mean difference: 74.9 (SD) seconds) and in each age group. Younger, vs. older, participants completed both the MoCA and CAVIRE tasks in a shorter time. There was a greater variability in the completion time for the MoCA, most markedly in the oldest group, whereas completion time was less variable for the CAVIRE tasks in all age groups, with most consistency in the 45–54 year-age group.

**Conclusion:** We demonstrate almost equivalent completion times for a VR and a questionnaire-based cognition assessment, with inter-age group variation in VR completion time synonymous to that in conventional screening methods. The CAVIRE has the potential to be an alternative screening modality for cognition in the primary care setting.

## Introduction

Dementia is a syndrome of one’s cognitive function deterioration, leading to increasing difficulties in coping with everyday activities. It is of increasing global concern. The World Health Organization projects that 50 million people worldwide live with dementia, with nearly 10 million cases diagnosed each year. The number of people with dementia is expected to triple by 2050 (World Health Organisation, 2020).

Singapore faces increasing prevalence of dementia in an aging population. According to the Well-being of the Singapore Elderly (WiSE) study (Subramaniam et al., 2015), one in ten people aged 60 years and above may have dementia. This translates to almost 82,000 people in 2018 and is expected to exceed 187,000 by 2050 (Alzheimer’s Disease Association Singapore, 2020). Early detection of cognitive impairment becomes imperative to initiate management, and also to prepare caregivers in handling the syndrome.

Identifying the early stages of cognitive decline remains challenging. No single cognitive screening tool is universally recommended for use worldwide. Screening tests commonly used in clinical practice include neuropsychological questionnaire-based assessments such as the Montreal Cognitive Assessment (MoCA) and the Mini-Mental State Examination (MMSE) (Abd Razak et al., 2019). These neuropsychological tests are rigorous and over-emphasize learning and memory dysfunction over other cognitive domains, such as executive function (Cullen et al., 2007) and perceptual-motor function (Alegret et al., 2009), which are more evident in early stages of dementia (Gauthier et al., 2006). These tests are also dependent on age, language and literacy status of the person. Exploring all cognitive domains via neuropsychological tests and quantifying overall cognitive performance together with history from a collateral informant is time-consuming. Such laborious process limits cognitive assessment within a primary care physician’s practice (Sabbagh et al., 2020). Developing a cognitive assessment modality that can objectively evaluate a person’s cognitive function across all six cognitive domains is thus necessary. Ideally the alternative test should be accomplished within an acceptably short time, address the limits of current paper-and-pencil tests, and contextualize and culturally adapt to the population in which it is being used.

Virtual reality (VR) has been used extensively in medicine, including for cognitive assessment, rehabilitation, and training (Pensieri and Pennacchini, 2014). By wearing a head-mounted device coupled with a device to detect hand gestures, VR enables a person to be immersed in a simulated environment that represents everyday life, allowing active interaction and participation of the subject within a realistic virtual environment. VR can potentially save manpower and time resources to complete the traditional questionnaire-based tests. It eliminates word literacy which impacts performance during cognitive assessment. Immersive virtual reality also provides a sensitive, ecologically valid way to assess cognition in a safe environment (Bohil et al., 2011), allowing an individual to interact with a non-threatening, controlled, yet realistic environment, for assessment of performance of day-to-day activities not otherwise assessed through traditional paper-and-pencil tests. It allows for consistent execution of test stimuli and the automation facilitates computation of performance scores and outcome measures.

Kourtesis et al. (2021) had validated the Virtual Reality Everyday Assessment Lab (VR-EAL), as the first immersive VR neuropsychological battery devised to assess cognitive functions central to everyday functioning, which showed enhanced ecological validity and a pleasant testing experience, without inducing cybersickness in a group of 41 Edinburgh participants with a mean of 13.80 years of education, 44% of whom were gamers. The participants had reported that VR-EAL tasks were significantly more ecologically valid and pleasant than the paper-and-pencil neuropsychological battery, and a shorter administration time was needed.

In Singapore, a novel system capable of giving automated audio-visual instructions while individuals perform VR tasks that cover all six cognitive domains—perceptual-motor function, language, learning and memory, executive function, complex attention, and social cognition—has been developed for Asians living in urban settings (Lim et al., 2021). Known as the CAVIRE (Cognitive Assessment using VIrtual REality) system, it is developed to assess the cognition of community-dwelling, ambulatory, older multi-ethnic Asians living in densely populated housing estates in Singapore.

The CAVIRE system is designed as a fully immersive VR with a three-dimensional environment, which interacts with the user. By fully immersive, we mean the system has the technical capability through use of a high-end head-mounted device, of allowing a participant to perceive his physical body in a natural way with the inference that what is being perceived is his actual surroundings, and not a virtual environment (Slater and Sanchez-Vives, 2016). This gives rise to the subjective illusion of “being there” in the environment depicted by the VR display, and this specific feeling is referred to as “place illusion” (PI). Plausibility illusion (Psi) refers to the illusion that the scenario being depicted is actually occurring (Slater, 2009), when the virtual environment relates to the participant’s actions, allowing the user to believe the plausibility that events are actually happening (Slater and Sanchez-Vives, 2016). When both place illusion and plausibility illusion occur, participants will then respond realistically to virtual reality, emphasized in the guidelines (Kourtesis et al., 2020) in developing an ecologically valid neuropsychological assessment that necessitates genuine responses from the user.

The time to complete the VR tasks is a quality indicator of the performance of this novel tool. It provides insight into the resources and hence the cost required to operationalize the system. Cognitively-healthy younger individuals are postulated to achieve better VR performance scores and shorter completion time due to exposure and familiarity with advanced technology compared to their more senior counterparts. The completion time for the MoCA is widely reported to be 10–15 min (Wong et al., 2015) in older adults but the time to complete it among younger Asian adults is not well established. Furthermore, MoCA has limitations, in terms of its ability to assess only specific cognitive domains, the need for trained healthcare personnel, and often requiring the subject to be literate. Understanding the completion time of a VR-based cognitive assessment in comparison with the MoCA across different age groups would be able to determine its utility potential and deployment in a time and manpower-constrained primary care practice.

## Aims

This is a feasibility study to compare the completion times of the standard neuropsychological questionnaire-based MoCA and the tasks in the fully immersive and automated CAVIRE in cognitively-healthy Asian adults aged between 35 and 74 years, overall, and in and across each 10-year age group (35–44; 45–54; 55–64; 65–74).

## Materials and Methods

This paper presents the results of the primary aim, the feasibility of using the CAVIRE^11^.

### Study Site

The study site was a public primary care clinic (polyclinic) situated in the southern region of Singapore. This polyclinic provides primary care healthcare services to an estimated population of 18,960 residents of varying Asian ethnicity in the Outram estate, of which 24.7% were aged 65 years and above in 2019 (Department of Statistics, Ministry of Trade and Industry, Republic of Singapore, 2019).

### Study Population

The participants were Asian patients who were attending medical consultations, or their accompanying persons or visitors at the polyclinic. The eligibility criteria included: (1) age between 35 and 74 years, (2) understood English (the medium of audio-visual instructions in the CAVIRE), (3) willing to complete the study questionnaires and undergo assessment using the CAVIRE, and (4) MoCA score of 26 or more.

Adults with any of the following were excluded: pre-existing diagnosis of cognitive impairment or dementia as self-reported or as documented in their electronic medical record; any disability which rendered them incapable of providing written informed consent; neurological deficits that might affect vision, hearing, speech or motor skills; or known motion sickness or epilepsy.

### Sample Size

25 participants were recruited in each 10-year age group: (A) 35–44, (B) 45–54, (C) 55–64, and (D) 65–74. The sample size was not estimated for the primary aim of this study, which assesses the feasibility of the CAVIRE. For feasibility studies, sample size justifications need to be provided but not necessarily a sample size calculation (Billingham et al., 2013). The Modified Wald method was utilized to compute the confidence interval of a proportion. By setting the proportion as 23 out of 25 participants in an age group, based on a one-sided 95% confidence interval, it can be assumed that at least 77% of future participants in that age group will be able to complete the CAVIRE assessment. A 90% completion rate (23 out of 25 participants in each age group) is deemed as adequate for the purposes of our feasibility study. Thus, in total, 100 cognitively-healthy participants, in four age groups of 25 participants each, were enrolled.

### Recruitment and Procedure During the Study Administration

A research assistant (RA) screened potential participants for eligibility at the waiting area of the polyclinic. After establishing their understanding of the study protocol and acquiring their written informed consent, the RA verified their diagnosis against their electronic medical records.

Participants then followed through the procedure, with the questionnaires completed first, then followed by the CAVIRE assessment, all within one sitting. The questionnaire gathered the participants’ details and their scores from the following validated assessment tools in the following sequence:

(1) Demographic data (age, gender, ethnicity, number of years of formal education)
(2) Abbreviated Mental Test (AMT)
(3) Barthel Index for Activities of Daily Living
(4) Lawton Instrumental Activities of Daily Living Scale
(5) Mini-Mental State Examination (MMSE)
(6) Montreal Cognitive Assessment (MoCA)
(7) CAVIRE VR assessment
(8) Participant feedback form

For the MoCA questionnaire, participants were provided with instructions, this taking 2–3 min, before the administration of the MoCA. While scoring the MoCA, an additional point correction was accorded to those with ≤10 years of education (Ng et al., 2013). Cognitively-healthy participants, defined as those with a MoCA score of 26 or higher were inducted into the next part of the study, which involved their completion of the CAVIRE assessment. Those who attained a MoCA score of less than 26 were excluded and referred for further clinical assessment at the polyclinic with their consent. The time to completion for the MoCA (start-time: when they start reading the first question of the MoCA questionnaire; end-time: completion of last question on the MoCA) was measured manually with a calibrated stopwatch (either: 1. VWR digital timer, one channel, Traceable^®^ Catalog Number VWRI609-0224, or 2. Casio Handheld Stopwatch Timer Model HS-3V-1R) and recorded in the research document. It excludes the time to transcribe the raw data from the questionnaire-based assessment into a person’s electronic medical records.

Participants were then briefed on the CAVIRE procedure and equipment. They sat on a chair and put on the VR head-mounted device (HTC VIVE Pro HMD set with Leap Motion mounted, with Lighthouse sensors and VIVE controllers), a new generation head-mounted device recognized for its superiority in pick-and-place, VR experience and interactive area, in line with technological recommendations for immersive VR research to safeguard the health and safety of the participants and the reliability of neuroscientific results (Kourtesis et al., 2019). They were inducted in a tutorial to a simulated virtual scenario, which took 3–5 min to complete. Participants continued to complete the 13 segments once they indicated readiness and ease in using the headset and familiarity with their hand movements in the VR environment. The time measured to complete the CAVIRE was recorded automatically in the program (start-time: beginning the task in Segment 1; end-time: completion of task in Segment 13).

The CAVIRE incorporates automated voice and visual instructions in English to guide the participants to complete the tasks. They performed these virtual tasks using hand gestures and head movements detected by motion sensors. Their speech was assessed using a voice recognition technology (English language) embedded in the system. Each segment features common everyday activities to assess specific domains of cognitive function through a person’s VR journey from an apartment to a grocery store:

1. Brushing and rinsing teeth
2. Preparing peanut butter bread for breakfast
3. Identifying pictures of important persons in the newspaper
4. Watching television, while listening to the weather forecast regarding impending rain on the radio
5. Naming the fruits in a shopping list and remembering the fruits
6. Choosing the appropriate clothing to go for grocery shopping
7. Remembering to pick up the umbrella, before opening and locking the door to leave the home
8. Taking the lift to level 1 in an apartment block by pressing the correct buttons
9. Looking to the left and right, and waiting for green pedestrian light, before crossing the street
10. Remembering and choosing the stipulated stall, i.e., the one which sells fruits
11. Picking the correct fruits based on recall from the shopping list (from Segment 5)
12. Calculating and paying the correct sum of money for all the fruits selected
13. Selecting the appropriate emotional response, with regards to scenes of a birthday party and car accident, respectively.

**FIGURE 1:**
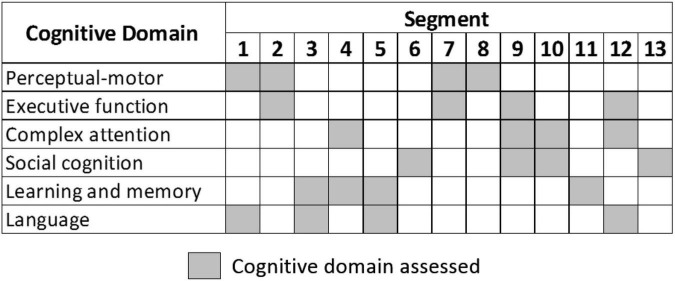
Cognitive domains assessed in the Cognitive Assessment by VIrtual REality (CAVIRE) segments.

These virtual segments cover the six cognitive domains as shown in Figure 1. Four segments (6, 8, 11, and 13) assess a single cognitive domain, while the tasks in the remaining nine segments evaluate two or more cognitive domains. To ensure a balanced evaluation framework, each cognitive domain is assessed over four different segments.

### Outcome Measures

An automated scoring and timing framework is embedded in the CAVIRE to assess each task performance and completion for the 13 segments. Each task is allocated a limited completion time, ranging between 30 and 90 s, and start time of CAVIRE is defined as when Task 1 is commenced. Each participant is allowed multiple attempts within the time limit, beyond which the participant will move on to the next task in an ordered sequence. The completion time and scores are computed automatically for each scenario and for the entire VR assessment. Participants who complained of headache, nausea and/or giddiness during the VR assessment were advised to discontinue, and considered as a dropout and reported in the results.

### Data Management and Monitoring

The data from the questionnaires were transcribed into the REDCap, a secure research database, and audited by a data management officer in the institution for errors. The VR data from the CAVIRE was exported to the same database and merged with the audited questionnaire data. The anonymized combined data were handed over to data analysts in the study team.

### Statistical Analysis

Potential confounders, including gender, ethnicity (Chinese, non-Chinese), education (secondary, post-secondary), and housing (public, private) as a surrogate for socio-economic status, were compared among age groups. Finding no significant difference among age groups for the confounders, the time to completion of MoCA and VR assessment indices were compared across the age groups using a one-way analysis of variance (ANOVA) *F*-test of the hypothesis H_0_: μ_1_ = μ_2_ = μ_3_ = μ_4_ (all means equal) vs. H_1_: μ_*i*_ ≠μ_*j*_ for at least one i ≠ j (i = j = 1, 2, 3, 4) (at least two means different). An *F*-test *p*-value of *p* < 0.05 was regarded as statistically significant for rejection of H_0_. *Post hoc* pair-wise comparisons among age groups were performed on time taken to complete the MoCA and CAVIRE tasks. Reported pair-wise comparison *p*-values are unadjusted for multiple comparisons given that the omnibus *F*-test provides protection against inflation of the false-positive error rate resulting from indiscriminate pair-wise testing. However, as an added measure of protection, a Bonferroni corrected significance level for six pair-wise comparisons was calculated as 0.05/6 = 0.0083 and applied. A test for a linear trend across the four ordinal age groups was tested using a contrast. Normality of residuals was assessed visually using linearized Q-Q plots and found to be tenable. The statistical analyses were performed using the SAS software v9.4.

### Ethics Approval

The SingHealth Centralized Institutional Review Board of Singapore approved the study (CIRB Reference Number: 2019/2782).

## Results

The study commenced recruitment in October 2020 and was completed by January 2021. One participant from the 35–44-years age group failed to complete the study due to apprehension during the administration of the MoCA questionnaire, constituting a dropout rate of 1%. It was not related to the VR performance.

Demographic characteristics of the remaining 99 participants are presented in Table 1, and did not differ significantly across the four age groups, labeled as Group (1) for 35–44 years; Group (2) for 45–54 years; Group (3) for 55–64 years and Group (4) for 65–74 years.

**TABLE 1:** Demographic characteristic frequency counts (%) and comparison among age groups.

**Demographic characteristic**		**Overall (*n* = 99)**	**Age group (years) count (%)**				
			**Group (1) 35–44 (*n* = 24)**	**Group (2) 45–54 (*n* = 25)**	**Group (3) 55–64 (*n* = 25)**	**Group (4) 65–74 (*n* = 25)**	***p*-value**
**Gender**	**Male**	44 (44.4)	12 (27.3)	11 (25.0)	12 (27.3)	9 (20.5)	0.765
	**Female**	55 (55.6)	12 (21.8)	14 (25.5)	13 (23.6)	16 (29.1)	
**Ethnicity**	**Chinese**	77 (77.8)	19 (24.7)	16 (20.8)	21 (27.3)	21 (27.3)	0.273
	**Non-Chinese**	22 (22.2)	5 (22.7)	9 (40.9)	4 (18.2)	4 (18.2)	
**Education**	**Up to secondary**	28 (28.3)	2 (7.1)	8 (28.6)	8 (28.6)	10 (35.7)	0.081
	**Post-secondary/tertiary**	71 (71.7)	22 (31.0)	17 (23.9)	17 (23.9)	15 (21.1)	
**Socio-economic status**	**Public housing**	66 (66.7)	17 (25.8)	16 (24.2)	19 (28.8)	14 (21.2)	0.470
	**Private housing**	33 (33.3)	7 (21.2)	9 (27.3)	6 (18.2)	11 (33.3)	

Table 2 summarizes the mean (SD) total time taken (in seconds) for each age group and the inter-age group mean total time differences. The time taken to complete the CAVIRE tasks as compared to the MoCA was significantly shorter, overall (*p* < 0.001) (mean difference: 74.9 (SD) seconds) and in each age group (*p* ≤ 0.01). Overall, younger participants completed both the MoCA and the CAVIRE tasks in a shorter time compared to the older participants, and the younger they were, the shorter the time of completion for both modalities of assessment. The Groups (1) vs. (2) and (3) vs. (4) pair-wise differences for time to completion for both MoCA and CAVIRE tasks were not statistically significant, but when compared between Groups (2) and (3), significant time differences were observed (Bonferroni significance level, *p* ≤ 0.0083). CAVIRE vs. MoCA time differences among age groups did not achieve significance (*p* = 0.147).

**TABLE 2:** Total time taken (SD) for MoCA and the CAVIRE tasks by age group with differences and 95% confidence intervals.

**Method**	**Age group Mean (SD) total time taken (sec)**					***P*-values: *F*-test (Linear trend)**	**Pair-wise differences between age groups 95% confidence interval on difference and *p*-value**					
	**Overall group (35–74 year)**	**(1) 35–44 year (*n* = 24)**	**(2) 45–54 year (*n* = 25)**	**(3) 55–64 year (*n* = 25)**	**(4) 65–74 year (*n* = 25)**		**(1) vs. (2)**	**(1) vs. (3)**	**(1) vs. (4)**	**(2) vs. (3)**	**(2) vs. (4)**	**(3) vs. (4)**
**MoCA**	515.12 (105.33)	476.7 (56.07)	455.3 (87.37)	559.2 (72.25)	567.8 (138.99)	< 0.0001 (<0.0001)	21.4 (–32.1, 74.9) 0.428	–82.5 (–136.0, –29.0) 0.003	–91.1 (–144.5, –37.6) 0.001	–103.9 (–156.9, –51.0) < 0.001	–112.5 (–165.4, –59.5) < 0.001	–8.56 (–61.5, 44.4) 0.7489
**The CAVIRE**	440.18 (68.47)	404.2 (55.71)	408.5 (52.3)	457.6 (57.67)	489 (70.47)	<0.0001 (<0.0001)	–4.35 (–38.1, 29.4) 0.798	–53.4 (–87.1, –19.7) 0.002	–84.9 (–118.6, –51.1) < 0.001	–49.0 (–82.4, –15.6) 0.004	–80.5 (–113.9, –47.1) < 0.001	–31.5 (–64.9, 1.92) 0.064
**Difference (95% CI) *P*-value**	74.94 (58.10, 91.78) < 0.001	72.5 (38.7, 106.3) <0.001	46.8 (13.6, 79.9) 0.006	101.6 (68.5, 134.8) <0.001	78.7 (45.6, 111.8) <0.001	0.1471 (0.3320)	25.8 (–21.5, 73.1) 0.282	–29.1 (–76.4, 18.2) 0.225	–6.18 (–53.5, 41.1) 0.796	–54.9 (–101.7, –8.05) 0.022	–32.0 (–78.8, 14.9) 0.179	22.9 (–23.9, 69.7) 0.334

*The Bonferroni corrected significance level for the six pair-wise comparisons is 0.05/6 = 0.0083.*

*Main effect means (SD) for Method: MoCA = 515.1 (105.3), VR = 440.2 (68.5); p-value < 0.0001; Mean difference (SD): 74.9 (84.5).*

*Main effect for Age Category (means averaged across methods not relevant), p-value < 0.0001.*

*Method × Age Category interaction (means in above table), p-value = 0.1471.*

All analysis methods were parametric and assumed normal error distributions. The assumption of normality of ANOVA residuals was assessed and found to be tenable. All comparisons were performed in the context of parametric 1-way ANOVA using *F*-tests followed by *t*-tests for pair-wise comparisons.

**FIGURE 2:**
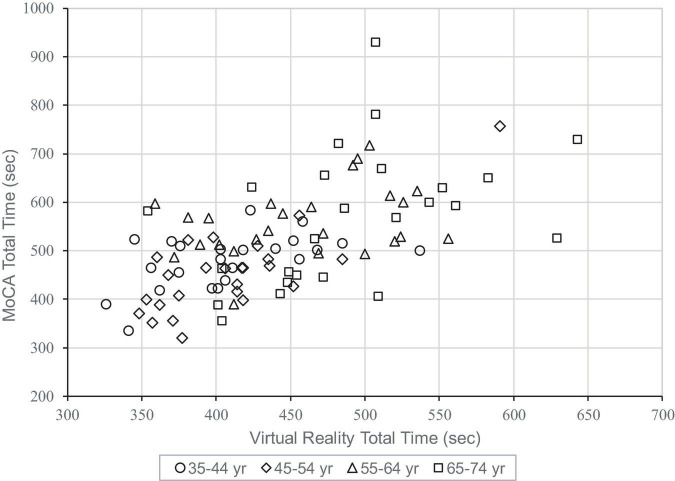
Scatter plot of Montreal Cognitive Assessment (MoCA) vs. Virtual Reality (CAVIRE) total time taken.

Figure 2 shows a scatter plot of the total time taken for the MoCA vs. the CAVIRE tasks, which further reflects that most participants across the different age groups took a shorter time to complete the CAVIRE tasks as compared to the MoCA, and younger participants generally performed better and more consistently. More variability in completion time was seen in Group (4).

**FIGURE 3:**
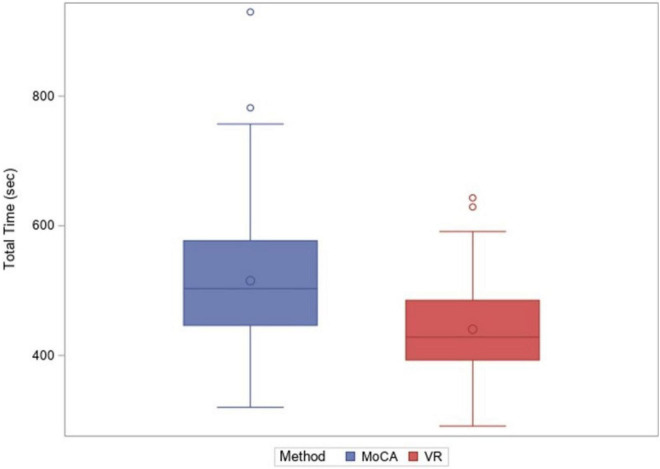
Total time taken (sec), MoCA vs. Virtual Reality (CAVIRE), overall.

**FIGURE 4:**
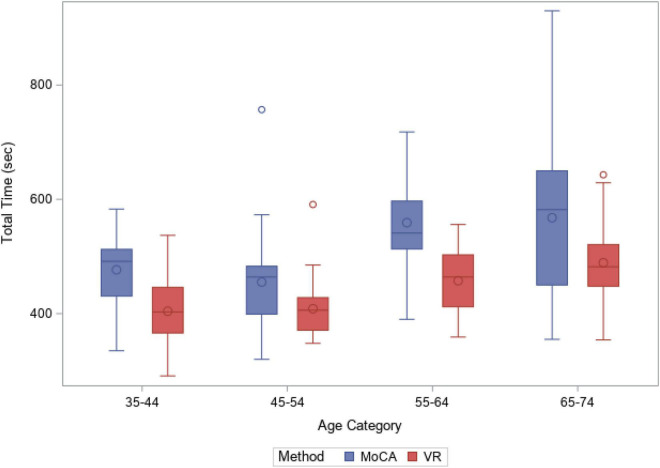
Total time taken (sec), MoCA vs. Virtual Reality (CAVIRE), by age groups.

Figures 3, 4 show the completion time for the MoCA and the CAVIRE tasks using a box-and-whisker plot. Figure 3 shows the overall completion time for MoCA and the CAVIRE for all groups, with the mean completion time (represented as the dot maker within each box) for MoCA as 515.12 s, and the mean completion time of CAVIRE as 440.18 s, excluding the outliers (dots at the top of the box-and-whisker plot). The median time is represented as the line within each box, and the top and bottom lines denote the minimum and maximum values recorded, with the interquartile range denoted by the height of the box.

Figure 4 reflects the completion time for MoCA and CAVIRE by age groups. The median time taken to complete the MoCA and the CAVIRE tasks was comparable in Groups (1) and (2). In addition, across Groups (2), (3), and (4), there was a greater variability in the total time taken to complete the MoCA, most marked in Group (4), whereas completion time was less variable for the CAVIRE tasks across all age groups, with most consistency in Group (2).

**FIGURE 5:**
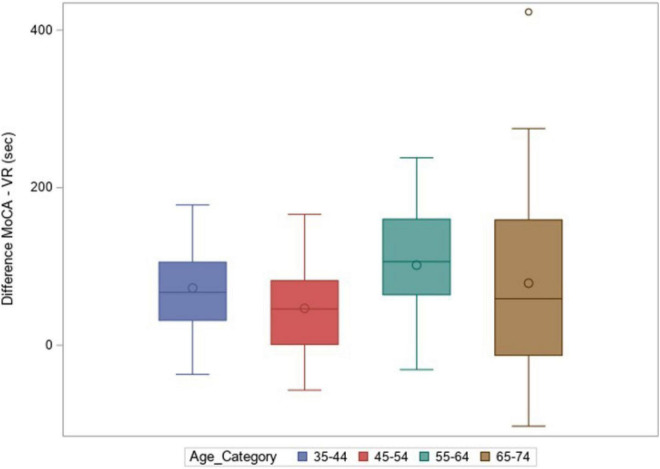
Difference [MoCA—VR (CAVIRE)] in total time taken (sec).

Figure 5 shows the difference in mean completion time between the MoCA and the CAVIRE tasks across age groups, again using a box-and-whisker plot. The difference was statistically significant across all age groups (*p* < 0.01) (Table 2).

**FIGURE 6:**
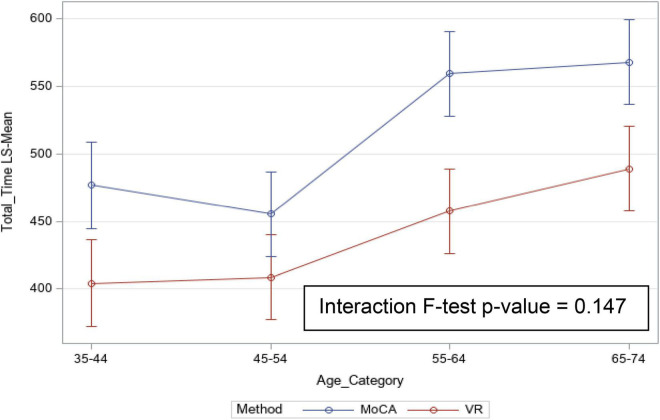
Mean (95% CI) total time taken (sec) for MoCA and VR (CAVIRE).

Figure 6 depicts the mean total time taken for the MoCA and the CAVIRE tasks, with the increase in completion of each modality statistically significant as one gets older, the steepest difference being where participants cross from Group (3) to Group (4). Completion time of the MoCA is not differentiated from Group (3) to Group (4), reaching somewhat of a plateau, but showed a difference for the CAVIRE tasks between Group (3) and Group (4), though not statistically significant (Table 2).

## Discussion

This study showed significant differentiation of the VR-based CAVIRE completion time vs. the MoCA completion time between the younger and older cognitively-normal Asian adults. The completion time for the CAVIRE tasks was shorter than the MoCA in each of four considered age groups, with the difference (MoCA—CAVIRE tasks) ranging from 48.6 s [Group (2)] to 101.6 s [Group (3)] (Table 2 and Figure 6). Additionally, the data from the questionnaire-based MoCA assessment has to be manually transcribed into a person’s electronic medical records. In contrast, the embedded automated scoring and time-recording in the CAVIRE eliminates such manual entry and increases convenience to the healthcare worker. While the time saving of about 1–2 min may not seem remarkable for an individual end-user, it can potentially be impactful in a healthcare establishment with high patient volume and limited healthcare manpower. Any non-clinical person can be trained to operate the CAVIRE, which will alleviate the medical or nursing resources in busy primary care practices. In contrast, the MoCA assessment usually requires a trained medical or nursing staff to administer the questionnaire, in order to maintain test validity, reduce variability and ensure high accuracy (MoCA Clinic and Institute, MoCA Test Inc., 2020), as recommended by its founder.

The results show wider intra-age group variability in time performance using the MoCA assessment. There was no overall time limit for completion of the MoCA, with the only time-limited component under the Language < Fluency—Name as many animals as possible in 1 min >, which already exists as part of the validated questionnaire. The VR modality reveals more consistency in its completion time (Figure 4) partly due to the automated system and standardized assessment procedure, which sets a maximum total time of 640 s for completion of the segments’ tasks, with an additional 100 s (hence total time of 740 s) to include instructions time and transition time between the segments, embedded in the CAVIRE system Without a forced time limit for each task, the participant could have performed better in terms of accuracy through repetition of the task.

Younger participants, in Group (1) and (2), completed both the MoCA and the CAVIRE tasks faster than their older counterparts (Figure 2). Their kinesthetic ability and processing speed may enable them to complete the CAVIRE tasks in a shorter time and with fewer repeated attempts. It is also possible that with each consecutive segment, study participants would have learned to control the depth and grasp of their hand motions to complete the tasks, and hence improve the time efficiency for task-completion in the later segments, albeit a different task and/or cognitive domain. This “real-time feedback” learning capability appears to be associated with better working memory performance. Miyake and Shah alluded that the younger adults were more adaptive to the mechanisms or processes involved in the control, regulation, and active maintenance of task-relevant information in complex cognition compared to the older adults (Miyake and Shah, 1999).

The difference in time-based performance was more apparent between the 45–54 and 55–64 year-age groups (Table 2 and Figure 6). Rieck et al. (2017) had reported that older-age adults (55–69 years) exhibited diminished brain activations with increasing task difficulty as compared to middle-age adults (35–54 years). Lower level of deactivation was observed between the transition from younger adults (20–34 years) to middle-age adults. This suggests that normal aging itself reduces neural efficacy and cognitive reserve, in task performance for accuracy and efficiency, and that as one moves into the fifth decade, there is a likelihood for a reduction in neural processing translating to diminished physical execution.

The significant decline in cognitive performance appears to be at the turn of 50 years, with the greatest mean time difference between the MoCA—VR in the 55–64 year-age group or Group (3). When assessed by the MoCA, the difference between Group (3) and (4) is minimal (Table 2 and Figure 6). However, the decline is sharper from Group (3) to Group (4) (Figure 6) when assessed using the VR modality. While the difference is not statistically significant, it suggests that VR may be a more discriminating tool than MoCA in detecting subtle cognitive performance impairments between the 55–64 and 65–74 year-age groups. This may allow earlier detection of subtle cognitive impairment in specific domains better assessed by VR, which is not otherwise observed through the use of conventional questionnaire-based tools.

## Strengths and Limitations

This study is novel to demonstrate almost equivalent time-based performance between a VR and a questionnaire-based cognition assessment. The opportunity of scalability lies in the VR system being able to assess an individual’s cognition as long as a person can see and hear even if he or she may not be able to write, eliminating the dependence on word literacy, although we recognize that Tasks 8 and 12 do need numeracy.

The study also shows the acceptability of senior participants in using the headset and interacting in the virtual environment without apprehension of the technology nor experiencing adverse effects related to the VR motions. Participants completed a 10-question feedback form at the end of the CAVIRE assessment, which assessed their level of comfort in using VR, level of similarity of VR compared to real world, level of interest toward VR, and overall experience, with positive responses. All of them completed the VR assessment, without describing cybersickness symptomatology such as nausea, headache or giddiness. We acknowledge that a more comprehensive and specific assessment of virtual reality-induced symptoms and effects (VRISE) and user experience through tools such as the Fast Motion Sickness Score (FMS) for assessing VRISE, and Virtual Reality Neuroscience Questionnaire (VRNQ) for assessing user experience and game mechanics, would be important in future studies determining the suitability of the CAVIRE tool as a neuropsychological tool for research and clinical purposes (Somrak et al., 2021). Interestingly, one younger participant failed to complete the study due to concern during the MoCA assessment, as part of the overall study protocol.

The study has its limitations. The limited number of 25 participants in each of the four designated age cut-off groups restricts its generalizability to the general population. Cognitively-healthy participants were identified using a single MoCA cut-off score of 26, and the study had not adjusted for other confounders such as intellectual ability, current and previous vocations (which may relate to exposure to VR technology), primary linguistic capability in processing the system’s audio-visual instructions, physical agility, and intra-age group variations in VR performance. However, no significant basic demographic difference was observed in the participants across the age groups.

Each VR segment has a pre-determined maximum completion time, which was based on an estimate of the time taken to complete a real-life task. When the time runs out in that particular segment, the participant is automatically moved to the next segment. The time-difference can be artificially narrowed between the best-performing participant vs. the least-performing individual due to the pre-set cap.

The shorter time needed to complete the VR assessment compared to MoCA is potentially cost-saving to the institution. However, the cost of investing to procure the VR equipment, their maintenance and upgrading of the VR system have to be computed to evaluate its eventual cost-effectiveness of using VR as an alternative assessment tool for cognition. Additional cost will be incurred to ensure infection control of the headset amidst the current COVID-19 pandemic.

## Conclusion

In summary, the VR-based CAVIRE has potential to be an alternative screening modality for cognition in a time-constrained primary care setting. It has demonstrated almost equivalent completion time across all age groups, and has been deployable with time-efficiency in the primary care practice. Younger adults completed the assessment in a shorter time than older adults, showing inter-age group variation, synonymous to conventional screening methods. There is potential to scale up its use as a cognition-screening modality.

## Data Availability Statement

The original contributions presented in the study are included in the article/supplementary material, further inquiries can be directed to the corresponding author/s.

## Ethics Statement

The studies involving human participants were reviewed and approved by the SingHealth Centralized Institutional Review Board of Singapore. The patients/participants provided their written informed consent to participate in this study.

## Author Contributions

JEL, WTW, JHMQ, and NCT designed the VR performance tasks, score matrix (as reflected in Table A1 in the Appendix), and the study protocol. RM reviewed the face validity of the 13 segments for cognitive assessment. TAT and SHL collaborated with FXMedia Internet Pte Ltd., the industrial collaborator to develop the VR system. WTW and NCT secured the funding and drafted the manuscript. The remaining authors reviewed and critiqued. All authors revised the draft, finalized and approved before submitting the manuscript.

## Conflict of Interest

The authors declare that the research was conducted in the absence of any commercial or financial relationships that could be construed as a potential conflict of interest.

## Publisher’s Note

All claims expressed in this article are solely those of the authors and do not necessarily represent those of their affiliated organizations, or those of the publisher, the editors and the reviewers. Any product that may be evaluated in this article, or claim that may be made by its manufacturer, is not guaranteed or endorsed by the publisher.

## Appendix

**TABLE A1:** Scoring algorithm of the VR assessment.

**Segment**	**Task**	**Score**					**Remarks**	**Cognitive Domain(s) assessed**	**Given time (sec)**
		**0**	**25**	**50**	**75**	**100**			
									
1	Following step-by-step instructions: (1) Squeeze toothpaste on toothbrush (2) Brush teeth (3) Rinse mouth	No attempt	Attempts, but unable to complete any Tasks	Complete 1 Task	Complete 2 Tasks	Complete all 3 Tasks	NIL	Perceptual motor Language	90
2	Preparing peanut butter bread without specific instructions: (1) Open peanut butter jar (2) Take peanut butter using knife (3) Spread peanut butter on bread	No attempt	Attempts, but unable to complete any Tasks	Complete 1 Task	Complete 2 Tasks	Complete all 3 Tasks	NIL	Perceptual-motor Executive function	90
3	Identify 3 images of important persons in the newspaper: (1) Lee Kuan Yew 2) Halimah Yacob 3) Goh Chok Tong	No attempt	Attempts, but unable to identify any images correctly	Identify 1 image correctly	Identify 2 images correctly	Identify all 3 images correctly	NIL	Learning and memory Language	30
4	(1) Remember to take umbrella before leaving the house later > > Television acts as a distractor > > Radio gives weather forecast of rain	NIL	NIL	NIL	NIL	NIL	This Task will be scored in Segment 7(b)	Complex attention Learning and memory	NA
5	(1) Name the 5 fruits by reading out aloud: Apple, Banana, Watermelon, Mango, Durian (2) Remember the 5 fruits	No attempt	Attempts, but unable to name any fruits correctly	Name 1–2 fruits correctly	Name 3–4 fruits correctly	Name 5 fruits correctly	Task #2 will be scored in Segment 11	Learning and memory Language	50
6	(1) Choose appropriate clothing (female/male) to go out for shopping	No attempt	Attempts, but unable to choose the correct clothing	Choose the correct clothing in 2 attempts or more	Choose the correct clothing in 1 attempt, with a time of 15 s or more	Choose the correct clothing in 1 attempt, with a time of less than 15 s	NIL	Social cognition	30
7(a)	(1) Open the door (2) Select the correct item to lock the door (3) Lock the door	No attempt	Attempts, but unable to complete any Tasks	Complete 1 Task	Complete 2 Tasks	Complete all 3 Tasks	NIL	Perceptual-motor Executive function	60
7(b)	Remember to take umbrella before leaving the house > > Hint given at two time points: (i) before locking the door (ii) after locking the door	Does not remember to take the umbrella at all	Remember to take the umbrella after closing the door outside the house (hint is given for the second time)	Remember to take the umbrella before closing the door outside the house (hint is given for the first time)	Remember to take the umbrella after opening the door inside the house (before any hints are given)	Remember to take the umbrella before opening the door inside the house (before any hints are given)	Continued from Segment 4	Continued from Segment 4	NA
8	(1) Press button to go down (outside lift) (2) Press button for Level 1 (inside lift)	No attempt	Attempts, but unable to complete any Tasks	Complete both Tasks in 2 attempts or more, respectively	Complete 1 Task in 1 attempt and Complete the other Task in 2 attempts or more	Complete Task #1 in 1 attempt and Complete Task #2 in 1 attempt	NIL	Perceptual-motor	20
9	(1) Press “Start” to cross after traffic light turns green (2) Looks to the left before crossing (3) Looks to the right before crossing	No attempt	Attempts, but unable to complete any Tasks	Complete 1 Task	Complete 2 Tasks	Complete all 3 Tasks	NIL	Executive function Complex attention Social cognition	60
10	Choose the correct store: i.e., clothes, electrical appliances, vegetables, fruits	No attempt	Attempts, but unable to choose the correct store	Choose the correct store in 2 attempts or more	Choose the correct store in 1 attempt, with a total time of 15 s or more	Choose the correct store in 1 attempt, with a total time of less than 15 s	NIL	Complex attention Social cognition	40
11	Choose the 5 fruits based on the previous shopping list: i.e., Apple, Banana, Watermelon, Mango, Durian	No attempt	Attempts, but unable to choose any fruits correctly	Choose 3 fruits or less correctly	Choose 4 fruits correctly	Choose 5 fruits correctly	Continued from Segment 5	Learning and memory	50
12	Following step-by-step instructions: (1) Calculate total price of the 5 fruits (2) Pay exact amount of money	No attempt	Attempts, but unable to complete any Tasks	Complete both Tasks in 2 attempts or more, respectively	Complete 1 Task in 1 attempt, and Complete the other Task in 2 attempts or more	Complete Task #1 in 1 attempt, and Complete Task #2 in 1 attempt	NIL	Executive function Complex attention Language	90
13	Choose the correct emotion with regards to the scene: (1) Birthday party (2) Car accident	No attempt	Attempts, but unable to complete any Tasks	Complete both Tasks in 2 attempts or more, respectively	Complete 1 Task in 1 attempt, and Complete the other Task in 2 attempts or more	Complete Task #1 in 1 attempt, and Complete Task #2 in 1 attempt	NIL	Social cognition	30
									
